# Perceived Everyday Discrimination and C- Reactive Protein Influence on Cognition of Older Black Adults

**DOI:** 10.1093/geroni/igab046.3718

**Published:** 2021-12-17

**Authors:** Sydney Kirven, Heather Farmer, Amy Thierry

**Affiliations:** 1 Xavier University of Louisiana, New Orleans, Louisiana, United States; 2 University of Delaware, Newark, Delaware, United States

## Abstract

Black adults and women are more likely to experience serious cognitive decline in older age than their white and male counterparts. Evidence suggests perceived discrimination is associated with poor cognition in older adults, though the mechanisms remain unclear. Perceived discrimination has been linked to elevated inflammatory markers, such as C-reactive protein (CRP), which increases risk for worse cognitive functioning. Yet, little research has investigated whether CRP is implicated in the association between discrimination and cognition among Black older adults or if this relationship differs by gender. Using 2006-2016 data from Black adults ≥65 years old(N=1343) in the nationally representative Health and Retirement Study, random effects linear regression models (1) tested the association between discrimination and cognitive functioning; (2) explored whether this relationship differed for women and men; and (3) assessed whether elevated CRP mediated the association between discrimination and cognitive functioning. More frequent discrimination was associated with worse cognitive functioning (b= -0.24, SE=0.11, p<0.05), though gender did not moderate this relationship. Elevated CRP was significantly associated with worse cognitive functioning (b= 0.40, SE=0.18, p<0.05). Discrimination remained statistically significant in this model, indicating no mediation by CRP. Of note, inclusion of depressive symptoms and cardiometabolic conditions accounted for the association between both discrimination and CRP with cognitive functioning. These findings demonstrate the need for more within-group research on older Black adults documenting the complex relationship between discrimination, inflammation, and cognitive health. This approach will provide greater understanding of the biopsychosocial mechanisms underlying disparities in cognitive functioning in Black adults.

